# Lentiviral vectors express chondroitinase ABC in cortical projections and promote sprouting of injured corticospinal axons

**DOI:** 10.1016/j.jneumeth.2011.08.003

**Published:** 2011-09-30

**Authors:** Rong-Rong Zhao, Elizabeth M. Muir, João Nuno Alves, Hannah Rickman, Anna Y. Allan, Jessica C. Kwok, Kasper C.D. Roet, Joost Verhaagen, Bernard L. Schneider, Jean-Charles Bensadoun, Sherif G. Ahmed, Rafael J. Yáñez-Muñoz, Roger J. Keynes, James W. Fawcett, John H. Rogers

**Affiliations:** a Cambridge Centre for Brain Repair, Forvie Site, Robinson Way, Cambridge CB2 0PY, UK; b Department of Physiology Development and Neuroscience, University of Cambridge, Downing St., Cambridge CB2 3EG, UK; c Laboratory for Neuroregeneration, Netherlands Institute for Neuroscience, Meibergdreef 47, 1105BA Amsterdam, The Netherlands; d Brain Mind Institute, Ecole Polytechnique Fédérale de Lausanne, 1015 Lausanne, Switzerland; e School of Biological Sciences, Royal Holloway, University of London, Egham, Surrey TW20 0EX, UK

**Keywords:** BDA, biotinylated dextran amine, CMV, cytomegalovirus, CNS, central nervous system, CS, chondroitin sulfate, CSPG, chondroitin sulfate proteoglycan, CST, corticospinal tract, DMEM, Dulbecco's modified Eagle's medium, GAG, glycosaminoglycan, LV-ChABC, lentiviral vector encoding chondroitinase ABC, PBS, phosphate buffered saline, PGK, phosphoglycerate kinase, Lentiviral vectors, Chondroitinase ABC, Corticospinal tract, Spinal cord injury, Axon regeneration

## Abstract

Several diseases and injuries of the central nervous system could potentially be treated by delivery of an enzyme, which might most effectively be achieved by gene therapy. In particular, the bacterial enzyme chondroitinase ABC is beneficial in animal models of spinal cord injury. We have adapted the chondroitinase gene so that it can direct secretion of active chondroitinase from mammalian cells, and inserted it into lentiviral vectors. When injected into adult rat brain, these vectors lead to extensive secretion of chondroitinase, both locally and from long-distance axon projections, with activity persisting for more than 4 weeks. In animals which received a simultaneous lesion of the corticospinal tract, the vector reduced axonal die-back and promoted sprouting and short-range regeneration of corticospinal axons. The same beneficial effects on damaged corticospinal axons were observed in animals which received the chondroitinase lentiviral vector directly into the vicinity of a spinal cord lesion.

## Highlights

► Lentiviral vectors can transduce neurons and glia to secrete chondroitinase. ► The active enzyme is secreted from long-distance axon projections from the cerebral cortex. ► Chondroitinase transduction promotes preservation and sprouting of damaged corticospinal axons.

## 1 Introduction

For several diseases and injuries of the central nervous system (CNS), potential forms of treatment would involve delivery of an enzyme, which might most effectively be achieved by a gene therapy approach. One example is the bacterial enzyme chondroitinase ABC, which is under consideration as a possible treatment for spinal cord injury. Because of the potential risks of prolonged or repeated infusions, we wished to adapt the chondroitinase gene so that it could direct secretion of active chondroitinase from mammalian cells, and to deliver it using lentiviral vectors so that both neurons and glia could secrete the enzyme.

The aim in using chondroitinase is to remove chondroitin sulfate proteoglycans (CSPGs), one of the major classes of inhibitory molecule present in the scar that forms after injury and prevents regeneration of damaged axons (Kwok et al., 2008). CSPGs consist of a core protein with attached glycosaminoglycan (GAG) chains. After CNS injury, CSPGs are synthesized by glial cells and create a barrier impenetrable for axon regeneration. CSPGs also exist in the uninjured CNS, particularly in matrix structures found around the cell body and neurites of some neurons, known as perineuronal nets. Perineuronal nets have a role in restricting plasticity; removing CSPGs has been shown to restore CNS plasticity.

Chondroitinase ABC is a bacterial enzyme from *Proteus vulgaris*, which degrades CSPGs by cleaving GAG chains, which are responsible for most of the inhibitory effect. Injection of chondroitinase into the brain or spinal cord promotes circuit plasticity, due to sprouting of axons and remodelling of intact circuits. Also, following spinal cord injury, chondroitinase treatment can promote long-distance regrowth of axons. Both local sprouting and long-distance regeneration are accompanied by functional recovery (Bradbury et al., 2002; Kwok et al., 2008; Bradbury and Carter, 2011).

Delivery of the enzyme can entail practical problems. The half-life of the enzyme at 37 °C can vary considerably. Although it can reach 2–3 weeks with stabilizers such as albumin or trehalose (Chau et al., 2004; Lee et al., 2010), it is much shorter in the absence of such agents, and the half-life was only 6 days in injected rat brain (Lin et al., 2008). A single injection can produce functional recovery by local axon growth and circuit reorganization (Galtrey et al., 2007; Cafferty et al., 2008), but longer-range regrowth of axons is likely to need more prolonged treatment, by chronic infusion or repeated injections, as has been given in experiments where long-distance tracts were severed (Bradbury and Carter, 2011). For eventual treatment of human patients, given the much larger size of the spinal cord and of the injured region, and the greater importance of the corticospinal tract, chondroitinase activity would probably be required over several months. Chronic delivery carries risks of tissue damage, inflammation, and infection.

Gene delivery of chondroitinase to the CNS could have several advantages. A long-lasting effect could be achieved with only a single injection, and any side-effects of injection, including the risk of causing further trauma and infection, would be minimized. Thus it could represent a powerful approach for promoting regeneration of the spinal cord after injury.

However, achieving secretion of a bacterial protein by mammalian cells can be problematic. As the newly synthesized chondroitinase polypeptide passes through the eukaryotic secretion pathway, cryptic *N*-glycosylation sites can become glycosylated, and because the bacterial sequence is not adapted for the eukaryotic pathway, this process can interfere with folding and secretion of the protein. We found that this process led to failure of functional enzyme production from the original bacterial chondroitinase gene when transfected into mammalian cells (Muir et al., 2010). We therefore modified the chondroitinase gene by directed mutagenesis of up to 6 selected *N*-glycosylation sites which mapped to positions important for structure or ligand binding. These mutations did not impair enzyme activity *in vitro*, and they allowed the translated protein to pass through the secretion pathway unimpaired, resulting in efficient secretion of active chondroitinase from transfected cells *in vitro* (Muir et al., 2010).

For greater efficiency of expression *in vivo*, we have now inserted the modified chondroitinase gene into three lentiviral vectors. Lentiviral vectors have been shown to transduce both neurons and glia efficiently in the CNS (Naldini et al., 1996b; Bloemer et al., 1997; Baekelandt et al., 2002; Zhao et al., 2003; Hendriks et al., 2007). In brain, expression is mainly in glia and leukocytes initially (Baekelandt et al., 2002), but also occurs in neurons from 2 weeks onwards (Bloemer et al., 1997; Baekelandt et al., 2002). In spinal cord, injections into dorsal column white matter (Zhao et al., 2003) or into post-injury glial scar (Hendriks et al., 2007) transduce all classes of glia, as well as many neurons in the surrounding gray matter, for ≥14 days. Expression is sustained in some populations of neurons for up to a year, and there is minimal immune response (Bloemer et al., 1997; Baekelandt et al., 2002). Such vectors have been used successfully to deliver therapeutic transgenes to the rodent CNS in models of neurological disease (e.g. Dull et al., 1998; Yáñez-Muñoz et al., 2006). Lentiviral vectors are thus the present vector of choice for high-level long-term local expression of a transgene, both in glia which produce the axon-inhibitory CSPGs, and in neurons which are affected by them.

Here we report that cells transduced with these vectors secrete active chondroitinase at high levels in tissue culture and in rat brain, and that the chondroitinase action in a model of spinal cord injury protects corticospinal axons from die-back and promotes sprouting. These results suggest that lentiviral vector-mediated expression of modified chondroitinase could form a useful component of a future treatment for spinal cord injury.

## 2 Materials and methods

### 2.1 Chondroitinase gene

Previous studies *in vitro* and in tissue culture (Muir et al., 2010) were used to design an optimized chondroitinase gene for secretion of enzyme from mammalian cells. The best gene from those studies was Y133, with 5 mutations, but we also found that mutation N751Q (present in Y133) was irrelevant as this residue is not glycosylated, whereas mutation N338Q (present in a different construct) appeared to have a slight extra beneficial effect (data not shown). We therefore synthesized gene Y1330 with the mutations N282K, N338Q, N345Q, S517A, N675Q (S517A abolishes glycosylation at N515), with the coding sequence replaced with preferred mammalian codons (Eurofins MWG Operon, Germany). (The full sequence is in Supplementary Data.) The signal sequence was from mouse matrix metalloproteinase 2 (Muir et al., 2010). This gene was cloned into vector pcDNA3.1 and was confirmed to produce highly active chondroitinase by *in vitro* transcription/translation and enzyme assay (data not shown).

### 2.2 Lentiviral vectors

The optimized chondroitinase gene was inserted into three lentiviral vectors (Table 1). The vectors are collectively referred to as LV-ChABC, and individually named LV-C and LV-D (with the cytomegalovirus immediate-early [CMV] promoter) and LV-P (with the mouse phosphoglycerate kinase [PGK] promoter for more long-term expression in neurons). All the vectors are integrating self-inactivating vectors and pseudotyped with VSV-G. The vectors were made by standard techniques, after first subcloning the chondroitinase transgene into a transfer plasmid such that the transgene could be transcribed into a packageable RNA upon transfection into HEK293T cells along with non-recombining plasmids that express lentiviral and VSV-G genes (Naldini et al., 1996a; Dull et al., 1998).

Each transfer plasmid specifies a vector RNA which contains, from 5′ to 3′, the RU5 fragment of the long terminal repeat (LTR), HIV-1 packaging signal in a Gag gene fragment, Rev response element, central polypurine tract/central termination sequence, CMV or PGK promoter with transgene, woodchuck hepatitis virus post-transcriptional regulatory element, and self-inactivating (SIN) 3′ LTR. The vector RNAs are essentially identical except for the promoter, polylinker and transgene. Vectors LV-C and LV-D were both created with the transfer plasmid pRRL (Dull et al., 1998), containing the CMV promoter, with the chondroitinase transgene inserted between the XbaI and NheI sites. Vector particles were generated by cotransfection of HEK293T cells with the transfer plasmid and two other plasmids (for LV-C: the VSV-G envelope protein vector pMD.G.2 and the viral core packaging construct pCMVΔR8.74; Dull et al., 1998) or three other plasmids (for LV-D: Rev encoded on a separate plasmid; Dull et al., 1998). Vector LV-P was created with a transfer plasmid derived from pRRL via the SIN-W-PGK vector, with the CMV promoter replaced with the PGK promoter and different polylinkers which includes an additional polypurine tract, and vector particles were generated by cotransfection with this plus two other plasmids (Déglon et al., 2000). Thus, LV-C and LV-P were produced with a second-generation system and LV-D with a third-generation system.

Viral particles were concentrated by ultracentrifugation and the viral particle-containing pellet was resuspended in 0.1 M phosphate-buffered saline pH 7.4 (PBS) or in Dulbecco's modified Eagle's medium (DMEM), and stored at −80 °C until further use. Lentiviral vectors were titered by a p24 antigen ELISA assay (Perkin Elmer), or by quantitative PCR (Yáñez-Muñoz et al., 2006), and then by determining the transgene-expressing units on either HEK293T cells or SCTM41 cells (Table 1).

### 2.3 Cell culture and transduction

Cell lines and primary astrocytes were as in Muir et al. (2010). To provide conditioned medium containing CSPGs as a substrate for secreted chondroitinase, just-confluent Neu7 cells were maintained in DMEM with Insulin/transferrin/selenium supplement (ITS^3+^; Sigma) for 48 h, and the medium collected. Otherwise, all cells were grown in DMEM containing 10% fetal bovine serum plus standard concentrations of penicillin, streptomycin and fungazone.

For transduction with LV-ChABC, cells were passaged the previous day onto 35-mm dishes so as to be ∼50% to 70% confluent when LV was added. LV was added (0.2–2.5 μl of stock for titration, 1.0 μl ≈ 6–10 × 10^5^ transducing units for western blots), diluted in a small volume of medium plus polybrene to 8 μg/ml. The medium was changed after 24 h. After another 24 h, it was replaced with serum-free Neu7 conditioned medium. This was collected after 24 h, centrifuged to remove detached cells, and concentrated 5- to 10-fold by centrifugation in a Centricon-50 unit (Millipore), mixed with protease inhibitor cocktail (Sigma P8340), and frozen for subsequent electrophoresis. Meanwhile the cells were fixed with 4% paraformaldehyde for immunocytochemistry.

Fixed cells were ‘blocked’ with PBS/2% sheep serum/0.3% Triton X-100 for 2 h, then immunocytochemistry was performed with rabbit anti-chondroitinase ABC (Acorda Inc., 1:2000, pre-absorbed by incubation with conditioned medium from Neu7 cells for 3 h at room temperature). After washing with PBS, peroxidase-linked goat anti-(rabbit Ig) (Vector Labs) was added at 1:200 in PBS/1% sheep serum/0.1% Triton X-100 for 1–2 h. After further washes, positive cells were visualized either by diaminobenzidine staining, or by tyramide signal amplification (1% Tyramide-488 green in amplification buffer plus 0.0015% H_2_O_2_ for 5 min). Some dishes were subsequently counterstained with bisbenzimide (Hoechst-33258) to visualize all cell nuclei. Cell counts of diaminobenzidine-stained cells were performed using a light microscope on >4 randomly selected 1-mm^2^ fields per dish.

Western blots were performed as in Muir et al. (2010). Antibodies were: mouse anti-NG2 (Santa Cruz sc33666 = mcAb 132.38), diluted 1:1000; mouse anti-‘stub’ (Seikagaku, mcAb 1B5, 1:250); rabbit anti-chondroitinase ABC (Acorda Inc., 1:2000, pre-absorbed as above). The anti-‘stub’ antibodies 1B5 and 2B6 sensitively detect products of chondroitinase activity, as they recognize disaccharide units (1B5: Δ-di-0S; 2B6: Δ-di-4S) that remain attached to the CSPG core proteins after chondroitinase ABC has cleaved off most of the GAG chains.

### 2.4 Animal surgery, tract tracing, and immunohistochemistry

#### 2.4.1 Animal surgery

Male adult Lister Hooded rats aged 3 months from Charles River were housed in groups in standard cages under a 12 h:12 h light/dark cycle, with food and water ad libitum. All procedures were performed in accordance with the UK Animals (Scientific Procedures) Act (1986). All surgical procedures were performed under inhalation anesthetic isoflurane (1–2% in a mixture of 25% nitric oxide and 50% oxygen). Body temperature was maintained at 37 °C during surgery using a heating pad.

#### 2.4.2 Cortical injection

Rats were placed in a stereotactic head frame, and the bregma was exposed as reference. One hole was drilled through the skull, and vector was injected into the left cortex (coordinates: AP, −0.5 mm; ML, −2.0 mm; DV, +1.5 mm). Volume injected was 1.0 μl (LV-P being diluted 2-fold) ≈5–8 × 10^5^ transducing units; saline was injected in controls. No obvious ill effects attributable to vector injection were observed. Expression was examined at 2, 4 and 8 weeks. All data are from at least 3 rats unless otherwise stated.

#### 2.4.3 Spinal cord lesion

The spinal cord was exposed with a laminectomy at level C4, a small slit was made on the dura, and a pair of fine forceps (Fine Science Tool, #11253-20) was positioned on either side of the dorsal columns and pushed down vertically into the cord for 1.5 mm. The forceps were then held tightly together for 20 s before being raised back out of the cord creating a crush lesion of the dorsal columns. In animals given concurrent operations, the spinal cord lesion was done ∼15 min before the cortical injection of vector.

#### 2.4.4 Intraspinal injection

In an additional series of animals, LV-C was injected into the spinal cord lesion region instead of into the brain. Immediately after the spinal cord lesion, two injections of 1 μl of either LV-C or surgical saline were carried out, 1 mm below and 1 mm above the lesion site. The injections were made through a pulled glass capillary (borosilicate thin wall with O.D. 1.0 mm and I.D. 0.78 mm, Harvard Instruments BS4 30-0039) of which the tip end that enters the spinal cord has a diameter of ∼20 μm. The opposite end was connected to 30 cm of polyethylene tubing (I.D., 0.40 mm) which was backfilled with mineral oil (Sigma) and linked to a syringe driven by a microdrive pump (Syringe Infusion Pump 22, Harvard Apparatus, Kent, UK). The glass capillary was first lowered vertically for 2 mm then raised for 0.5 mm, then infusion started and lasted for 5 min. Infusion rate was set at 200 nl/min. The capillary was then left to stand in place for another 3 min before being lifted, to allow complete absorption.

#### 2.4.5 Anterograde axon tract tracing

The corticospinal tract (CST) was traced with cortically injected biotinylated dextran amine (BDA). The BDA injection was wider than the original LV-ChABC injection to ensure good uptake, which might be impeded by glial scarring in the original injection track. About 2 weeks before termination of experiments, the animals were deeply anesthetized with isoflurane and secured in a stereotaxic frame. With a dental drill, three holes were made in the skull over the forelimb representation in the sensorimotor cortex, and 1 μl of 10% BDA (MW 10,000, Molecular Probes, in 10 mM PBS) was injected slowly using a 26-gauge Hamilton syringe at a depth of 1.5 mm at each site. (Stereotaxic coordinates: AP +0.5, ML −2; AP −0.5, ML −3; AP −0.5, ML −1.8.) After the last injection, the skin was sutured and the animals were returned to standard housing conditions for 14–19 days.

#### 2.4.6 Tissue preparation

Animals were sacrificed by sodium pentobarbital overdose (200 mg/kg intraperitoneally), and transcardially perfused with PBS followed by 4% paraformaldehyde in 0.1 M phosphate buffer. The brains and spinal cords were then post-fixed in the same fixative overnight at 4 °C, followed by 30% sucrose in phosphate buffer overnight at 4 °C. Tissue was frozen in optimized cutting agent (OCT) before cutting. For brain tissue, and spinal cord at C1, coronal sections 40 μm thick were cut with a freezing microtome and kept free-floating at 4 °C. Spinal cord below C1 was cut in parasagittal or horizontal plane at 30 μm.

#### 2.4.7 Fluorescent immunostaining

Tissue sections were stained with antibodies 2B6 (as above) or CS56 (Sigma; 1:400), or *Wisteria floribunda* agglutinin (WFA; Sigma; 1:150). Sections were ‘blocked’ for 1 h with TBST (Tris-buffered saline with 0.1% Triton-X100) plus 10% goat serum, then 200–300 μl primary antibody at appropriate dilution in blocking buffer was added to each well and the plates were gently shaken overnight at 4 °C. Sections were then washed in TBST for 3× 30 min at RT before secondary antibodies were added to wells for 2 h at RT. For BDA, final detection was with Streptavidin Alexa Fluor 488 conjugate (Invitrogen). After further washes, sections were mounted onto 1% gelatin coated glass slides, covered with FluorSave™ mounting medium (Merck, #345787), and coverslip.

#### 2.4.8 Quantification of axon sprouting, retraction, and regeneration

Axon sprouting was quantified by drawing 6 lines in the gray matter, parallel and 100 μm apart, from 1.0 mm to 1.5 mm rostral to the lesion front (the rostral edge of the lesion cavity), as shown in Fig. 5a. The number of axons that crossed each line was counted on 4–7 sections per animal; the number of crossings of all 6 lines were summed as the total sprouting number; the axon number in a transverse section at C1, ∼2 mm rostral to the lesion, was counted as the total labeled axon number (to account for the inter-animal BDA tracing variability); and sprouting number was normalized against the total labeled axon number and the number of sections to give the axon sprouting index. To quantify retraction and regeneration, 10 lines were drawn transversely (Fig. 5a), and the number of traced axons crossing each line was counted, and calculated as the percentage of the total labeled axon number. The same procedures were used for animals with LV injected into brain (Fig. 5) or spinal cord (Fig. 6). Controls with saline injections in either location showed indistinguishable axon distributions and were therefore combined for analysis. Significance was assessed by two-tailed *t*-tests.

### 2.5 Enzyme activity assay

Animals received LV-ChABC injection to cerebral cortex as above. After 2 or 4 weeks they were sacrificed by sodium pentobarbital overdose, and a 3-mm cube of cortex from the injected area, and an equivalent sample from the contralateral side, were dissected and frozen and subsequently homogenized in enzyme buffer for total enzyme activity assay (Lin et al., 2008). Polyacrylamide discs of 3.5 mm diameter, each containing 7.5 μg of chondroitin sulfate (CS-A, Sigma), were incubated with the homogenates for 1 h at 37 °C. A range of standard quantities of commercial chondroitinase ABC (Seikagaku) was used as a positive control. (One unit generates one micromole of unsaturated disaccharide from chondroitin-6-sulfate per minute at 37 °C, pH8.0.) After incubation, the discs were rinsed briefly with water, fixed in 40% methanol:8% acetic acid, and stained with 0.2% Alcian blue 8GX (Sigma) for 30 min. Discs were then examined for loss of color due to the digestion of CS relative to the negative control.

## 3 Results

### 3.1 Validation of lentiviral vectors in tissue culture

**Fig. 1:**
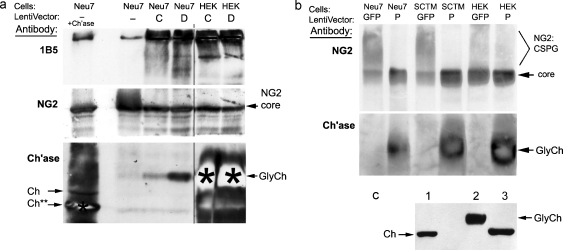
Western blots showing that active chondroitinase is secreted following transduction of tissue culture cells with the lentiviral vectors. Neu7 conditioned medium (a source of CSPGs) was placed on transfected cells for 24 h, then analyzed by western blotting. (a) LV-C and LV-D. The first two lanes are positive and negative controls with Neu7 medium not exposed to transfected cells; lane 1 was digested *in vitro* with commercial chondroitinase (Sigma, 20 mU, 37°, 3 h). (Top panel) Probed for carbohydrate ‘stub’ epitope produced by chondroitinase action (antibody 1B5). Neu7 conditioned medium (lane 2) shows little immunoreactivity, but incubation with cells after transduction with LV-ChABCs generates extensive reactivity. (Middle) Probed for NG2. Undigested NG2 (lane 2) appears largely as a characteristic ‘smear’ above the core protein band due to the heterodispersed high-Mr GAG chains, and this is all converted to core protein by digestion with commercial chondroitinase (lane 1) or by incubation with cells after transduction with LV-ChABC. (Bottom) Probed for chondroitinase ABC. Commercial chondroitinase (lane 1) shows both full-length band (Ch) and a shorter band (Ch**) due to proteolytic activity during incubation with medium. LV-ChABC all generate a diffuse chondroitinase band (GlyCh: partially glycosylated): this migrates more slowly than bacterial chondroitinase, confirming that it has some glycosylation at sites which were not mutated. Some lanes are overexposed as the experiment was principally intended to detect digestion of CSPGs; also see (c). Black bar or star indicates intense bands which partially bleached during imaging. (b) LV-GFP (control) and LV-P. (Top) Probed for NG2; the high-Mr smear due to GAG chains (marked NG2:CSPG), whose distribution varies according to cell type, is all reduced to core protein following LV-P transduction. (Bottom) Probed for chondroitinase ABC. (c) Confirmation of chondroitinase secretion. (1) Commercial chondroitinase (Sigma); (2) medium from HEK cells transduced with LV-C; (3) the same after treatment with *N*-glycosidase to remove residual glycosylation.

**Fig. 2:**
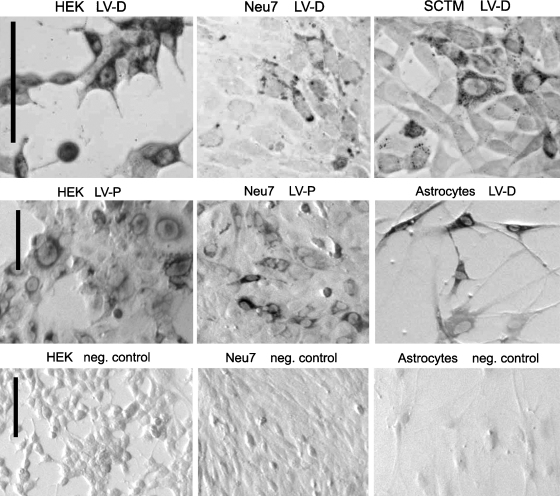
Immunocytochemistry showing expression of the vectors in tissue culture cells. Cells were transduced with lentiviral vectors, fixed, reacted with antibody against chondroitinase, and visualized with peroxidase diaminobenzidine reaction. Scale bars, 100 μm. Top row: LV-D in HEK293T cells, Neu7 cells, and SCTM41 cells. Middle rowLV-P in HEK293T cells, Neu7 cells, and primary astrocytes. Bottom row: negative control cultures with no vector.

To investigate whether the lentiviral vectors could successfully transfect cells and secrete functional chondroitinase enzyme, we used them to transduce several cell lines and primary astrocyte cultures. Chondroitinase production was assessed by western blotting (Fig. 1) and immunocytochemistry (Fig. 2).

For western blotting, 2 days after adding vector, the medium was replaced with conditioned medium from Neu7 cells, which contains abundant CSPGs to serve as a substrate. This medium was collected 24 h later and chondroitinase activity was assessed by western blots showing degradation of CSPGs (Fig. 1):

(i) by degradation of NG2 proteoglycan;
(ii) by production of disaccharide ‘stub’ immunoreactivity (antibody 1B5);
(iii) by direct detection with antibody against chondroitinase ABC.

All three vectors showed successful expression in HEK293T cells, rat glial cell lines, and primary astrocytes. The NG2 and 1B5 antibodies showed essentially complete digestion of the glycosaminoglycans of the CSPGs in each sample. The chondroitinase antibody showed that the amount of secreted protein was considerably greater than with earlier experiments using plasmid transfection (Muir et al., 2010). It was especially prolific in HEK293T cell cultures transduced with LV-C or LV-D, which employ the CMV promoter. The amount of chondroitinase seen on a western blot from LV-C-transduced HEK293T cells was equivalent to ∼1 mU of commercial chondroitinase per 5 μl of medium (Fig. 1c).

Transduction was also visualized directly by immunocytochemistry with antibody against chondroitinase ABC (Fig. 2). Positive cells were clearly identified by stained cytoplasm contrasting with well-defined unstained nuclei. There was a wide range of intensities and it is possible that some cells secreting chondroitinase were not visualized, especially in the more confluent cultures. Therefore, the titres deduced from these cultures (Table 1) are considered to be lower limits. LV-P (with chondroitinase under the PGK promoter) gave similar high transduction efficiencies in several different cell types: HEK293T cells, Neu7 cells (astrocyte-derived), SCTM41 cells (Schwann cell-derived), and primary astrocytes. LV-C and LV-D (with the CMV promoter) also transduced all these cell types well but the titres were severalfold higher on HEK293T cells than the others. As expected with integrating lentiviral vectors, expression persisted after passaging the transduced cells.

### 3.2 Injection of lentiviral vectors into rat cortex produces chondroitinase activity locally and along axons

**Fig. 3:**
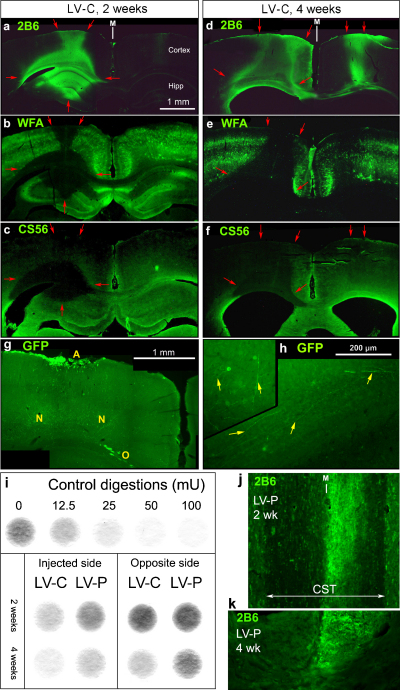
*In vivo* LV-ChABC injection into the rat cortex results in chondroitinase activity and the activity is also transported along axons. Injection was into deep layers of cortex on the left. Scale bar, 1 mm. (a–c) LV-C, 2 weeks: adjacent coronal sections from one brain stained for chondroitinase-produced stub (a), perineuronal net (b) and chondroitin sulfate (c). Chondroitinase digestion (area outlined with red arrows) is seen around the injection site and in the cortical white matter and adjacent hippocampus. On the original of (a), a faint column of reactivity can also be seen in the contralateral cortex. M, midline. (d–f) LV-C, 4 weeks: a similar series of sections, showing that chondroitinase digestion has extended along axon tracts and to the contralateral cortex. (g,h) LV-GFP, 4 weeks: similar sections from a brain injected with a lentivector encoding farnesyl-GFP; 4 weeks; stained with antibody against GFP. (g) Cortex near injection site, showing strong expression in astrocytes at surface (A), and oligodendrocytes in white matter/corpus callosum (O), and large numbers of neuronal fibers (N). (h) High-power views of contralateral side showing GFP-positive axons that have crossed in the corpus callosum and (inset) ascending in the cortex opposite the injection site. (i) Enzyme activity assay of functional chondroitinase level 4 weeks after LV injection into the cortex. Disks were loaded with equal amounts of chondroitin sulfate (CS) and each was incubated with extract of cortex from one brain to allow digestion by chondroitinase expressed in the sample. They were then stained for CS. Top row: incubated with extract of control brain (0), and with various amounts of commercial chondroitinase (12.5–100 mU). Bottom panels: incubated with extracts of LV-injected brains. All samples from the injected side gave some digestion, especially the 4-week samples, as did the 4-week samples from the opposite side. (j,k) 2B6 staining on spinal cord after LV-P cortical injection. (j) 2 weeks: horizontal section at level C3-C4. (k) 4 weeks: transverse section at level C1. M, midline. (For interpretation of the references to color in this figure legend, the reader is referred to the web version of this article.)

**Fig. 4:**
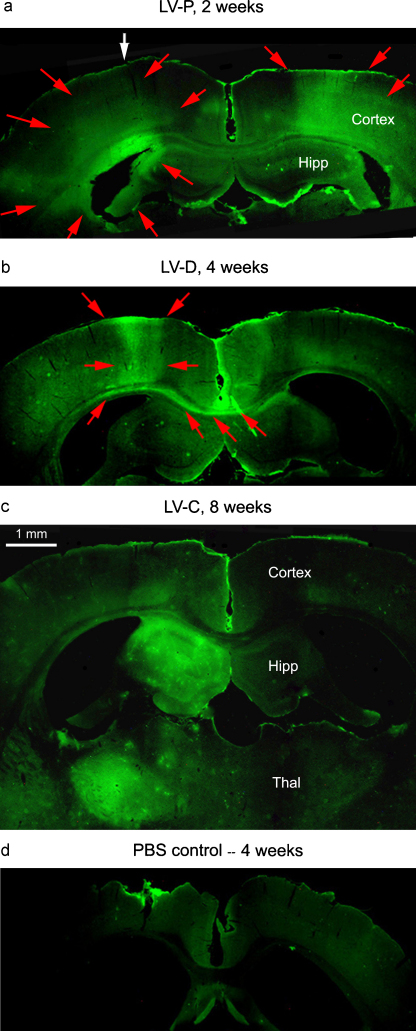
Expression of chondroitinase in rat brain from different LVs at different times: coronal sections stained with Ab-2B6 as in Fig. 3. Scale bar, 1 mm. (a) LV-P, 2 weeks: there is widespread digestion on both sides (red arrows). White arrow marks the approximate position of the injection. (b) LV-D, 4 weeks: more circumscribed digestion within ∼0.5 mm of the injection track, but also strong digestion within the corpus callosum extending to the contralateral side. (c) LV-C, 8 weeks: patchy digestion in various regions, especially ipsilateral hippocampus (Hipp) and thalamus (Thal). (d) PBS-injected control, 4 weeks: no specific immunoreactivity. (For interpretation of the references to color in this figure legend, the reader is referred to the web version of this article.)

To assess *in vivo* activity, each LV-ChABC was injected into the left cortex of adult rat brain, and the effects were examined at 2, 4 and 8 weeks, using antibody 2B6 to detect the carbohydrate ‘stub’ residue of chondroitinase digestion (Figs. 3 and 4). Similar results were seen with all three vectors and so they are described together, although the spatial extent of digestion was generally greatest with LV-P and least with LV-D. CSPG degradation was evident by 2 weeks (*n* = 5): the digestion region stained with Ab-2B6 spanned all cortical layers and part of the hippocampus (Fig. 3a). In the same region there was a strong reduction in chondroitin sulfate level detected by antibody CS56 staining (Fig. 3c), and elimination of the perineuronal nets whose main components are CSPGs linked to hyaluronan, as detected by *Wisteria* hemagglutinin (Fig. 3b). In a typical brain injected with 1 μl of LV-C, the digested region measured 3.5 mm in the anterior–posterior direction at 2 weeks. Four weeks post-injection (*n* = 6), the chondroitinase degradation zone stained with Ab-2B6 had spread laterally in the injected cortex, and was also present in the corpus callosum (cortical axon tract crossing the midline), and in the contralateral cortex in a column opposite the injection site (Fig. 3d). This extended distribution indicates secretion of chondroitinase from commissural axons. The degradation residue was still present by week 8 (*n* = 3) with even broader distribution but uneven and generally weaker (Fig. 4c), presumably due to a balance between induction, degradation, and resynthesis of CSPGs.

To test directly whether a lentiviral vector could lead to expression of its product in axons crossing to the contralateral cortex, an animal was injected with a lentiviral vector encoding farnesyl green fluorescent protein (GFP) into the same position of the cortex, and GFP expression was examined by immunohistochemistry at 4 weeks. GFP was expressed at high levels in glia around the injection track and in the cortical white matter, and also in numerous nerve fibers throughout the thickness of the cortex on the injected side (Fig. 3g). Some GFP-positive axons extended through the corpus callosum, and some were also seen ascending in the contralateral region of cortex, confirming that the transgene product can be distributed to that location by commissural axons (Fig. 3h). No 2B6 staining was present in the GFP-positive regions, confirming that the lentiviral vector itself does not induce CS degradation.

The enzyme activity level at 2 and 4 weeks was studied in one set of brains using CS digestion assay in gel circles (Lin et al., 2008). A 3-mm cube of cortex from the injected area and the equivalent volume from the contralateral side were dissected and frozen and subsequently homogenized for total enzyme activity assay. Lighter color indicates that the enzyme activity of that sample is stronger (Fig. 3i). LV-C already produced active enzyme at 2 weeks, and by week 4 the activity was also detectable in the contralateral cortex. LV-P produced a similar pattern but the activity level was lower. The total amount present in the LV-C-injected cortex at 4 weeks is estimated as ∼25 mU from this assay, and the distribution is consistent with the 2B6 immunostaining results.

Since CS degradation in brain was observed to have spread through the corpus callosum axons, we also looked for it in the corticospinal tract (CST) in two animals with LV-P injection into the left cortex. Sections of spinal cord stained with Ab-2B6 showed that degradation residue was present in the right CST, i.e. the tract arising from the injected area, and it extended down to C4 level (Fig. 3j and k). Specific 2B6 staining was not seen outside the CST.

### 3.3 Cortical injection of lentiviral vector enables axon preservation and sprouting after spinal cord injury

**Fig. 5:**
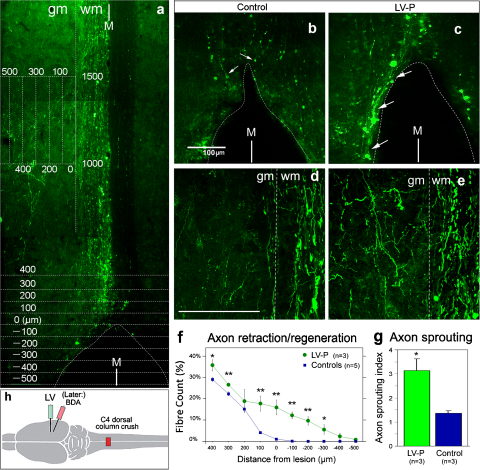
In animals given a spinal cord lesion, LV-ChABC injected into cerebral cortex enhances corticospinal axon growth towards the lesion and lateral axon sprouting. Horizontal sections of spinal cord at the level of the dorsal columns are shown, 4 weeks after lesion ± LV-P cortical injection, stained for BDA, which had been injected into cortex to trace the CST. Cranial at top; lesion cavity at bottom in (a–c). M, midline. Images are laterally inverted, so the right CST (labeled from the left cortex) is shown to the left. Scale is indicated in (a); scale bars 100 μm for other panels. (a) Methods to quantify retraction, regeneration and sprouting. Spinal cord at C3-C4 from a lesioned animal with no vector treatment. At bottom, grid for measuring longitudinal retraction/regeneration relative to the lesion front (the rostral edge of the lesion cavity). At top, grid for measuring lateral sprouting in gray matter (gm) relative to the edge of the white matter (wm). (b) Control spinal cord lesion without LV injection: axons have retracted from the cranial edge of the lesion cavity (retraction bulbs arrowed). (c) In animals which received LV-P cortical injection, axons grow along the lesion edge caudally (arrowed). (d) Spinal cord 1.0–1.5 mm above the lesion, just left of the midline, in a control lesioned animal; axons show little lateral sprouting. (e) Similar section in a LV-P-treated animal shows substantial lateral sprouting into the gray matter. (f) Measurements of longitudinal retraction/regeneration: percentage of axons which remain at various distances above and below the lesion front, for control and LV-treated CST (±SEM). Significance by *t*-test: *P < 0.05; **P < 0.01. (g) Measurements of lateral axon sprouting index (per section per 100 axons labeled, ±SD), for control and LV-treated CST. Significance, *P* < 0.05. (h) Diagram of the surgical procedures.

The effect of the LV-ChABC cortical injection on lesioned axons was studied with the CST of spinal cord injured rats (Fig. 5). A bilateral dorsal column crush lesion at C4 level was made, then LV-P, or saline in the control group, was injected unilaterally into the cortex during the same surgery (*n* = 3 per group). Subsequently, the axons were traced with cortical BDA injection, then animals were perfused at 4 weeks post-lesion. This operation totally severs the main tract of CST axons, in the dorsal columns, and a roughly oval cavity forms in the lesion area. Effects on the axons on the rostral side were evaluated by two criteria: the number of axons regenerated in the caudal direction, and the number of axons sprouted laterally into the gray matter. The method of quantification is illustrated in Fig. 5a. In the control saline-injected group, axons retracted rostrally for several hundred microns and formed retraction bulbs at the tips (Fig. 5b and f). In contrast, in the LV-P group, the axons had fewer retraction bulbs, and some extended along the edge of the cavity and grew caudally (Fig. 5c and f), reaching up to 500 μm along the edge. The difference in distribution was statistically significant (Fig. 5).

Axon sprouting laterally into the gray matter was also enhanced by the LV-P treatment. Compared to the saline control group, a larger number of axons crossed the gray matter/white matter boundary, and the fiber length within the gray matter was increased (Fig. 5d,e, and g). The sprouting index in the LV-P group was 3.15 ± 0.48 (SD), significantly more than that in the control group which was 1.32 ± 0.07 (*P* < 0.05).

### 3.4 Spinal cord injection of lentiviral vector after spinal cord injury produces similar improvements

We next asked whether local injection of LV-ChABC into the injured spinal cord, which would generate chondroitinase locally from transduced glia as well as possibly from neurons, would have similar effects to cortical injection, which delivered chondroitinase to the injury site specifically via the corticospinal axons. For this purpose, the same bilateral dorsal column crush lesion was made at C4 level, and immediately afterwards, two injections of LV-C were given at 1 mm caudal and 1 mm rostral to the lesion, totalling 1 μl (6 × 10^5^ units). Control animals were given the same lesion but received saline injections instead of vector. Subsequent staining with Ab-2B6 antibody confirmed that there was a wide zone of CSPG degradation product around the lesion in the LV-C injection group but not in control animals (data not shown).

**Fig. 6:**
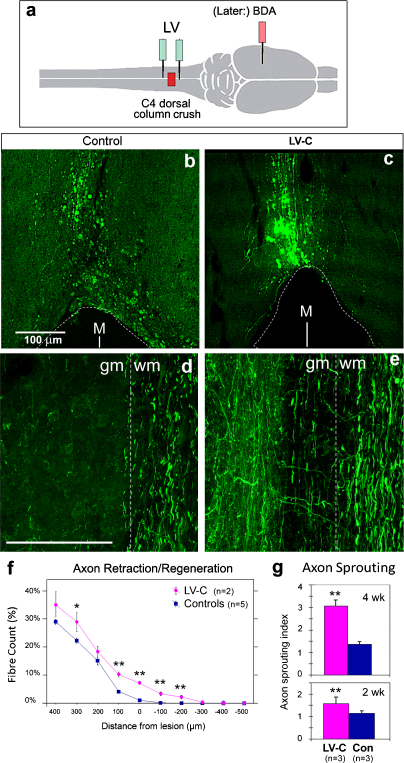
Intraspinal LV-ChABC injection promotes axon sprouting and growth in lesioned spinal cord. Procedures and illustration formats are as for Fig. 5, except that instead of LV-P injection into the brain, LV-C was injected into the spinal cord at the cranial and caudal edges of the lesion. (a) Diagram of the surgical procedures. (b–e) Horizontal sections of spinal cord, 4 weeks after lesion ± LV-C injection, stained for BDA, which had been injected into cortex to trace the CST. Cranial at top. M, midline; gm, gray matter; wm, white matter. Scale bars, 100 μm. (b) Control spinal cord lesion with saline injection: axons have retracted from the lesion front. (c) In a LV-C-treated animal, axons show little retraction, and some grow along the lesion edge. (d) Spinal cord 1.0–1.5 mm above the lesion in a control animal; axons show little lateral sprouting. (e) Robust axon lateral sprouting into the gray matter in a LV-C treated animal. (f) Measurements of longitudinal retraction/regeneration, at 4 weeks: percentage of axons which remain at various distances above and below the lesion front, for control and LV-treated CST (±SEM). Significance by *t*-test: *P < 0.05; **P < 0.01. (g) Measurements of lateral axon sprouting index (per section per 100 axons labeled, ±SD), for control and LV-treated CST, at 4 weeks (top) and in a separate group of animals at 2 weeks (bottom). Significance, *P* < 0.01.

The CST axons were traced with bilateral BDA cortical injection, animals were perfused 4 weeks post-lesion, and axon retraction and sprouting were examined as before (Figs. 5a and 6). The results were the same as with cortical LV-ChABC injection and spinal cord lesion. In the control group, axons had retracted from the lesion front, and retraction bulbs were found at the axon endings (Fig. 6b). With the LV-C injection, the CST axons were present right up to the cavity, and some extended a few hundred micrometers along the lesion edge past the lesion front (Fig. 6c). These fibers showed a tortuous shape instead of a smooth path, consistent with their being regenerated fibers. Local delivery of LV-C allowed more than 5% axon fibers to grow pass the lesion front and extended for up to 300 μm (Fig. 6f).

Local delivery of LV-C also significantly enhanced the lateral sprouting of axons above lesion. At 4 weeks, the sprouting index in the LV-C group was 3.06 ± 0.27 (SD), more than double that in the control group which was 1.36 ± 0.11 (Fig. 6g; *P* < 0.01). Another group of animals was operated in the same way and examined at 2 weeks, and they also showed significantly more sprouting in the LV-C group (1.58 ± 0.09) than the control group (1.14 ± 0.09; Fig. 6g; *P* < 0.01). The sprouting index at 2 weeks was lower than at 4 weeks, consistent with the axon sprouts continuing to grow during this period, while the chondroitinase gene is still being expressed.

Although lentiviral vectors generally produce few adverse effects, we wished to check whether the LV-ChABC vectors specifically produced inflammation following injection into the CNS. For this purpose, sections of spinal cord from several animals in each group were stained with antibody OX42, to visualize microglia and macrophages, and with antibody against GFAP, to visualize astrocytes (data not shown). The same pattern was seen in lesioned spinal cords that had been injected with saline (2 or 4 weeks, *n* = 4), or with LV-C (2 or 4 weeks, *n* = 3), or with LV-P (2 or 4 weeks, *n* = 3; these animals were not BDA traced). They showed that both microgliosis and astrocytosis were restricted to a zone ∼0.5 to 1 mm wide around the lesion edge, except in the dorsal columns rostrally (and occasionally, to a lesser extent, caudally) where microgliosis was observed for a long distance; this is normally seen in such lesioned animals due to the degeneration of myelinated axons damaged by the crush lesion. Thus, the extent of inflammation in the LV-ChABC injected animals was similar to or less than the inflammation in the controls.

## 4 Discussion

These experiments demonstrate that a combination of selective mutagenesis to ablate cryptic *N*-glycosylation sites, and expression in lentiviral vectors, allows the bacterial chondroitinase ABC enzyme to be copiously expressed in mammalian CNS.

Chondroitinase activity (as shown by the presence of 2B6 stub immunoreactivity) was detected in injected brains for up to 8 weeks, the longest time point assessed. This probably represented continuing production of active enzyme up to that time, as it extended well beyond the areas labeled at 4 weeks. Active enzyme was detected directly from brains at 4 weeks by enzyme activity assay. We estimate that one μl of vector (6–10 × 10^5^ transducing units) generated several tens of milliunits of enzyme in injected brain in this assay (Fig. 3g), and a similar quantity in conditioned medium of cultured glial cells after 3 days by western blots (Fig. 1).

Prior studies with lentiviral vectors in the CNS have shown that they transduce all types of glial cells and neurons, with neuronal expression predominating from 2 weeks onwards. In the case of the chondroitinase vectors, the pattern of chondroitinase digestion indicates that the enzyme is being secreted from neurons, because glial transduction could not account for the widespread chondroitinase activity, including in long-distance axonal projections: the corpus callosum and the corticospinal tract. It was not possible to visualize chondroitinase production directly as the antibody, which stained transduced subconfluent cells in tissue culture, did not stain transduced cells in over-confluent cultures nor in sections from injected brains. However, a GFP-transduced brain confirmed that a lentiviral vector can indeed express its product in the decussating axons that pass through the corpus callosum.

Lentiviral vectors do not themselves produce significant inflammation nor an immune response (Bloemer et al., 1997; Baekelandt et al., 2002), if a purified preparation is used, and we did not observe astrocytosis in the chondroitinase-expressing areas of brain beyond that attributable to the injection itself. Likewise in spinal cord, astrocytosis and microgliosis covered a region no greater than that seen in control lesioned spinal cords, the glial reactions being attributable to the crush lesion rather than the vector. There were also no obvious behavioral anomalies attributable to the vector injections.

The results in spinal cord injury can be compared with those of Bradbury et al. (2002), who performed the same spinal cord lesion followed by intermittent infusion of 300 mU of chondroitinase intrathecally over 10 days. Both treatments prevented retraction of the severed CST axons from the lesion (at the time-points assessed), and produced significant regeneration of CST axons along the edge of the lesion cavity, and lateral sprouting of CST into the gray matter. Thus, within the 4-week timescale of our axon tracing experiments, the results appear to be similar, whether the vector is delivered to the cortex or to the spinal cord.

Bradbury et al. (2002) also found more extensive regeneration of axons, for >4 mm, at 8–10 weeks, and this was accompanied by significant behavioral recovery. We are now undertaking longer-term experiments to directly compare lentiviral treatment with serial infusion, and to assess behavioral as well as anatomical recovery, and to test whether lentiviral vector delivery is equally (or more) effective if given some time after the lesion.

The present results indicate that delivery of chondroitinase by a lentiviral vector to the cerebral cortex generates substantial chondroitinase activity locally, and significant activity in long-range axon projections, attributable to enzyme transport down axons. In animals with lesioned CST, the enzyme has a beneficial effect on the severed axons that is comparable in effect to direct infusion, and likely to be much more suitable for therapeutic use. In contrast to injection or infusion, the lentiviral vector only has to be administered once; it confers greatly reduced risks of mechanical damage, inflammation, and infection at the infusion site; it generates enzyme continuously within the CNS tissue, including axon tracts; and it is effective for a long period. It can now be combined with other treatments in long-term animal models, to overcome other inhibitory agents in the injured CNS and to enhance the regenerative ability of neurons, in order to work towards a combination therapy suitable for human patients.

## Figures and Tables

**Table 1:** Lentiviral vectors encoding modified chondroitinase ABC.

Name	Promoter	Lab	Concentration^a^	Minimum titre^b^	References
LV-C	CMV	Amsterdam	99 μg/ml of P24	6 × 10^5^ TU/μl (HEK cells)	Naldini et al. (1996a), Hendriks et al. (2007),
LV-D	CMV	London	1.44 × 10^9^ gen/ml	8 × 10^5^ TU/μl (HEK cells)	Dull et al. (1998), Yáñez-Muñoz et al., 2006
LV-P	PGK	Lausanne	479 μg/ml of P24	∼10^6^ TU/μl (SCTM cells)	Déglon et al. (2000)

a gen: genomes, by Q-PCR.

b TU: transducing units. These titres are approximate values derived from the number of cells positive by chondroitinase immuno-staining 3 days after transduction of a subconfluent monolayer, quoted for the cell type which gave the highest titre.
