# Supplementary material for: Lentiviral vectors express chondroitinase ABC in cortical projections and promote sprouting of injured corticospinal axons
Source: J Neurosci Methods. 2011 Sep 30;201(1):228–38. doi: 10.1016/j.jneumeth.2011.08.003 (PMC3235548; doi:10.1016/j.jneumeth.2011.08.003)
Supplement: Supplementary file 1 [file mmc1.doc]

**Lentiviral vectors that express Chondroitinase ABC in cortical projections and promote sprouting of injured axons**

Rong-Rong Zhao, et al.

**SUPPLEMENTARY DATA:**

________________________________________________________________________

**Sequence of the synthetic optimised chondroitinase gene, Y1330.**

The encoded protein has the mutations N282K, N338Q, N345Q, S517A, N675Q

(S517A abolishes glycosylation at N515). It is similar to Y133 but not identical: unlike Y133, it encodes N338Q, but N751 is unchanged [reverted].

(We had evidence that N338Q made a slight improvement, whereas N751Q made

a slight impairment and this residue is not glycosylated.)

The codons are optimised for mammalian preferences.

In the translation below, mutated residues are in red, and the signal sequence is underlined.

_________________________________________________________________________

1 gcggccgcca tggaagcccg tgtagcgtgg ggagccctag caggcccttt gcgggtcctt

61 tgcgttctgt gctgtctact tggtcgtgcc atagcggcaa ctagtaatcc cgccttcgac

121 cccaaaaacc tgatgcagag cgaaatctac catttcgccc agaataatcc tctcgcagac

181 ttcagcagtg ataagaatag catcttgact ctgtccgaca agcggtcaat catgggcaat

241 cagagccttc tttggaagtg gaaagggggt tctagtttca ccctgcacaa gaaactgatt

301 gtacctaccg ataaagaggc cagcaaagcg tggggcagaa gtagcacacc ggtgttttcc

361 ttctggctct ataacgagaa acctatcgac ggttacctga caatcgattt tggggagaag

421 ctgatttcaa cctccgaagc acaagcgggc tttaaagtga agcttgactt cacaggatgg

481 agagccgtag gcgtctctct gaacaatgac ctggaaaata gagagatgac cctcaacgct

541 accaacacca gcagcgacgg tacacaggac tctattggca ggagtctggg agcaaaagtg

601 gatagcatac ggttcaaagc tcccagtaat gtctctcagg gcgagatcta tattgaccgc

661 ataatgttta gcgtcgatga cgcccgatac cagtggtctg actatcaggt gaagacacgg

721 ctatctgagc cagaaatcca gtttcacaac gtgaagcctc aactgcccgt tacaccagag

781 aacctggccg cgatcgacct cattcggcaa aggctcatca acgagtttgt gggtggagaa

841 aaagagacga atcttgccct cgaggagaag atctctaagc tgaaatccga ctttgatgca

901 cttaacatac acactttagc caatggcgga actcaggggc gacacctcat taccgataag

961 caaatcatca tctaccaacc agaaaacctc aattcccagg acaaacagct gttcgacaac

1021 tacgtgattt tgggacagta cactacactg atgttccaaa tttcccgagc ttacgtgctc

1081 gagaaagatc ctacgcagaa agcccagctg aaacagatgt acctgctgat gacaaaacat

1141 ctgctcgacc aggggttcgt caagggctct gctctggtta ctacacacca ctggggatac

1201 tcatcccgat ggtggtacat ctccacattg ctcatgtcag atgccctgaa ggaggcaaat

1261 ctgcagaccc aggtctatga ttccctgctg tggtatagta gggagttcaa gagttccttc

1321 gacatgaagg tatctgcaga ttccagcgat ctggactatt tcaatacgtt gtccagacag

1381 cacctggctc tgctgctcct agagccagac gaccaaaagc gcattaacct agtgaacacc

1441 ttttctcact atatcaccgg tgcacttact caagttcccc caggcggaaa agacgggctc

1501 agacccgatg gaactgcttg gcgccatgag ggcaactatc ccgggtactc atttcccgct

1561 ttcaagaatg ctgcccagtt gatttattta ctgcgggata ccccgttttc agtgggcgaa

1621 agtggatgga ataatctgaa aaaagctatg gtttccgctt ggatttacag caatccggaa

1681 gtgggcttac ctttggccgg ccgtcatccc tttaacagcc catctctgaa aagcgtggct

1741 cagggctact attggctagc tatgtcagcg aaatcctctc ccgataagac tttagcttcc

1801 atctatcttg ccataagcga caagacccag aatgagagta ccgccatctt tggagagact

1861 attacgccag catccctgcc tcaaggattc tatgccttta acggtggtgc ttttggcata

1921 cacaggtggc aggacaagat ggtcacactc aaggcctaca atactaatgt gtggtctagc

1981 gagatctaca acaaagacaa taggtatggt cgctaccaga gtcacggagt ggcccagata

2041 gtcagccaag gttcccagct tagccaggga taccaacagg aaggctggga ttggaaccgg

2101 atgcaggggg ccacgactat acaccttccc ttgaaggatc tggatagccc aaaaccgcat

2161 acactgatgc agaggggcga gaggggtttc tccggcactt caagcttaga agggcagtat

2221 ggcatgatgg cattcgacct gatctatcca gcgaatcttg agagatttga ccccaacttc

2281 accgcaaaga agagcgttct cgccgccgac aaccacctta tctttattgg gtctaacatc

2341 aattctagtg ataagaacaa aaatgtggaa actaccttgt ttcagcatgc cattacgccc

2401 accttgaata cactgtggat taatgggcag aagattgaaa atatgcccta ccagacgact

2461 ctgcaacagg gggattggtt aatcgacagt aacgggaatg gctacctaat cacgcaagcc

2521 gagaaggtaa acgtcagtcg ccaacatcag gtttcagcag aaaacaagaa caggcaacct

2581 acagaaggga acttttcatc agcatggata gaccatagca caagaccgaa agatgccagc

2641 tacgaataca tggtgttcct ggatgctact cccgagaaaa tgggagaaat ggcccagaag

2701 ttcagagaaa acaacgggtt gtatcaggtg ctccgcaaag ataaggacgt gcacattata

2761 ctcgacaagc tctcaaacgt tacaggatac gccttttacc agccagcttc tatcgaggat

2821 aagtggatca aaaaggtgaa taagccagca attgtgatga cacatcgcca aaaggatacc

2881 ttgatcgtgt ctgcagttac accagatttg aacatgaccc ggcagaaagc tgctacccct

2941 gtcacaatca acgtcaccat taatgggaag tggcagagcg ctgataagaa cagcgaagtg

3001 aagtatcagg taagcgggga taacactgag ctcaccttca cctcctattt cggcatccct

3061 caggagatta agttatcccc tctgccttga gaattc

**TRANSLATION:**

AAAMEARVAWGALAGPLRVLCVLCCLLGRAIAATSNPAFDPKNLMQSEIYHFAQNNPLAD

FSSDKNSILTLSDKRSIMGNQSLLWKWKGGSSFTLHKKLIVPTDKEASKAWGRSSTPVFS

FWLYNEKPIDGYLTIDFGEKLISTSEAQAGFKVKLDFTGWRAVGVSLNNDLENREMTLNA

TNTSSDGTQDSIGRSLGAKVDSIRFKAPSNVSQGEIYIDRIMFSVDDARYQWSDYQVKTR

LSEPEIQFHNVKPQLPVTPENLAAIDLIRQRLINEFVGGEKETNLALEEKISKLKSDFDA

LNIHTLANGGTQGRHLITDKQIIIYQPENLNSQDKQLFDNYVILGQYTTLMFQISRAYVL

EKDPTQKAQLKQMYLLMTKHLLDQGFVKGSALVTTHHWGYSSRWWYISTLLMSDALKEAN

LQTQVYDSLLWYSREFKSSFDMKVSADSSDLDYFNTLSRQHLALLLLEPDDQKRINLVNT

FSHYITGALTQVPPGGKDGLRPDGTAWRHEGNYPGYSFPAFKNAAQLIYLLRDTPFSVGE

SGWNNLKKAMVSAWIYSNPEVGLPLAGRHPFNSPSLKSVAQGYYWLAMSAKSSPDKTLAS

IYLAISDKTQNESTAIFGETITPASLPQGFYAFNGGAFGIHRWQDKMVTLKAYNTNVWSS

EIYNKDNRYGRYQSHGVAQIVSQGSQLSQGYQQEGWDWNRMQGATTIHLPLKDLDSPKPH

TLMQRGERGFSGTSSLEGQYGMMAFDLIYPANLERFDPNFTAKKSVLAADNHLIFIGSNI

NSSDKNKNVETTLFQHAITPTLNTLWINGQKIENMPYQTTLQQGDWLIDSNGNGYLITQA

EKVNVSRQHQVSAENKNRQPTEGNFSSAWIDHSTRPKDASYEYMVFLDATPEKMGEMAQK

FRENNGLYQVLRKDKDVHIILDKLSNVTGYAFYQPASIEDKWIKKVNKPAIVMTHRQKDT

LIVSAVTPDLNMTRQKAATPVTINVTINGKWQSADKNSEVKYQVSGDNTELTFTSYFGIP

QEIKLSPLP*EF
